# Cyclic AMP-Dependent Regulation of Kv7 Voltage-Gated Potassium Channels

**DOI:** 10.3389/fphys.2020.00727

**Published:** 2020-06-30

**Authors:** Jennifer van der Horst, Iain A. Greenwood, Thomas A. Jepps

**Affiliations:** ^1^Vascular Biology Group, Department of Biomedical Sciences, University of Copenhagen, Copenhagen, Denmark; ^2^Molecular and Clinical Sciences Institute, St. George’s University of London, London, United Kingdom

**Keywords:** Kv7 (KCNQ), cAMP, PKA, EPAC, physiology

## Abstract

Voltage-gated Kv7 potassium channels, encoded by *KCNQ* genes, have major physiological impacts cardiac myocytes, neurons, epithelial cells, and smooth muscle cells. Cyclic adenosine monophosphate (cAMP), a well-known intracellular secondary messenger, can activate numerous downstream effector proteins, generating downstream signaling pathways that regulate many functions in cells. A role for cAMP in ion channel regulation has been established, and recent findings show that cAMP signaling plays a role in Kv7 channel regulation. Although cAMP signaling is recognized to regulate Kv7 channels, the precise molecular mechanism behind the cAMP-dependent regulation of Kv7 channels is complex. This review will summarize recent research findings that support the mechanisms of cAMP-dependent regulation of Kv7 channels.

## cAMP-Dependent Signaling

### Regulators of cAMP: G Protein-Coupled Receptors

First discovered by Dr. Earl W. Sutherland in 1958 (Rall and Sutherland, 1958; Sutherland and Rall, 1958), cyclic adenosine monophosphate (cAMP) is well-recognized as an important intracellular secondary messenger molecule that can induce a cascade of events to influence cellular function (Patra and Brady, 2018). cAMP orchestrates numerous signal transduction pathways through the activation of several downstream effector proteins, resulting in a wide variety of cellular processes including gene transcription, cell growth and cell differentiation (Dumont et al., 1989; Sassone-Corsi, 1995; Mayr and Montminy, 2001; Sands and Palmer, 2008; Bacallao and Monje, 2015).

cAMP is created from ATP through the action of Adenylate cyclases (AC), of which there are nine membrane-bound isoforms (AC 1-9) and one soluble isoform (sASC). The characteristics of each isoform are summarized effectively in several reviews (Taussig and Gilman, 1995; Simonds, 1999; Hanoune and Defer, 2001; Sadana and Dessauer, 2009; Tresguerres et al., 2011; Halls and Cooper, 2017). Membrane-bound ACs are stimulated downstream of Gs-specific G protein-coupled receptor (GPCR) activation, while the sAC is insensitive to GPCR-dependent regulation (Braun and Dods, 1975; Braun et al., 1977; Forte et al., 1983). When a GPCR is activated by its extracellular ligand, it causes a conformational change in the receptor which activates the associated heteromeric G protein complex, consisting of an α, ß and γ subunit. Subsequently, the Gsα dissociates from the receptor and Gßγ subunits, activating AC and increasing cAMP levels (see Pierce et al., 2002 for a detailed review of this signaling pathway). ACs can also be regulated by other intracellular signals, including Ca^2+^ and PKC (see Halls and Cooper, 2017 for a detailed review).

Phosphodiesterase’s (PDEs) remain the only known route of cAMP degradation. The expression and localization of PDEs within a cell controls the magnitude and duration of cAMP-dependent events, as well as the compartmentalization and intracellular gradients of cAMP (Baillie, 2009). Thus, the expression and localization of PDEs in a cell contributes to the specificity of the cAMP-response following activation.

### Effectors of cAMP: PKA and EPAC

cAMP mediates its effect by activation of effector proteins including protein kinase A (PKA), Epac (Exchange protein directly activated by cAMP), or cyclic nucleotide-gated channels (CNGCs) (Shabb, 2001; Bos, 2006; Biel and Michalakis, 2009). For many years it was believed that the effects of cAMP were mediated exclusively through the activation of intracellular PKA. PKA is a heterotetrameric holoenzyme consisting of two regulatory (R) subunits and two catalytic (C) subunits (Krebs and Beavo, 1979; Taylor et al., 2013; Turnham and Scott, 2016). There are two types of regulatory subunits, RI and RII, each consisting of two isoforms, RIα, RIβ, RIIα, RIIβ, each having different tissue expression, subcellular localization and cAMP binding affinity (Bechtel et al., 1977; Corbin et al., 1977, 1978; Zoller et al., 1979; Rannels and Corbin, 1980; Ringheim and Taylor, 1990; Taylor et al., 2012; Weigand et al., 2017). PKA that contains either RI or RII, form the two classes of PKA termed type I or type II, respectively. Binding of four cAMP molecules to each R subunit induces a conformational change and activates the kinase, resulting in the free catalytic subunits phosphorylating serine and threonine residues in specific substrate proteins (Welch et al., 2010).

Scaffolding proteins, such as A Kinase Anchoring Proteins (AKAPs; reviewed in Rubin, 1994; Scott et al., 2013; Dema et al., 2015) bind to the PKA-R subunits at the dimerization/docking domain at the N-terminus and targets PKA to specific subcellular locations to ensure specificity in signal transduction by placing PKA close to its appropriate substrate target, allowing compartmentalization of PKA (Scott et al., 1990; Carr et al., 1991; Bauman et al., 2006; Gold et al., 2006). AKAPs also bind other proteins such as protein kinase C and PDEs (Moleschi and Melacini, 2014). The AKAP family has more than 50 members and can be classified according to their binding specificity for the PKA-R subunits (Wong and Scott, 2004). The majority of the AKAPs bind to PKA-RII, however, several AKAPs bind to PKA-RI (Kinderman et al., 2006; Sarma et al., 2010). Only a few AKAPs have dual-specificity and can bind to both PKA-RI and PKA-RII, although with lower affinity for PKA-RI (Burns et al., 2003). This lower affinity for PKA-RI is due to the structural difference in the dimerization/docking domain in the N-terminal of RI (Huang et al., 1997; Gold et al., 2006). AKAPs are expressed in different tissues of the body and can facilitate tissue-specific signaling in many cells types including neurons (Glantz et al., 1992; Moita et al., 2002; Dell’Acqua et al., 2006), heart (Fink et al., 2001; Marx et al., 2002; Ruehr et al., 2004), and pancreas (Lester et al., 1997).

cAMP also activates Epac (Exchange protein directly activated by cAMP) (de Rooij et al., 1998; Kawasaki et al., 1998). Epac proteins, stimulated by cAMP binding, activate the Ras superfamily of small GTPases, Rap1 and Rap2. There are two known Epac proteins, Epac1 and Epac2, which are both present in almost all tissues but have different expression levels (Roscioni et al., 2008). Epac acts as guanine-nucleotide exchange factors and catalyzes the exchange of GDP for GTP on Rap1 and Rap2 with subsequent activation of these two GTPases (Cheng et al., 2008). This leads to activation of further downstream pathways, which will result in a variety of cellular functions, depending on specific tissue and cell type. In cardiac myocytes, for example, Epac plays a role in the cardiac Ca^2+^ regulation by increasing Ca^2+^ release from the sarcoplasmic reticulum (SR) via ryanodine receptor 2 (RyR2) and thereby effecting contraction (Fujita et al., 2017). Furthermore, a signaling pathway in which cAMP acts via Epac to modulate ion channel function has been identified (Renström et al., 1997; Kang et al., 2006; Stott et al., 2016). Additionally, Epac enhances the synaptic release of neurotransmitters following cAMP elevation in neurons (Shariati et al., 2016).

Cyclic nucleotide-gated channels (CNGCs) are also activated directly by cAMP signaling (Ludwig et al., 1998; Santoro et al., 1998). CNGCs are non-selective cation channels expressed in many tissues, including the heart, lung, kidney, pancreas, liver, spleen, testis and various neuronal systems (Kaupp and Seifert, 2002; Biel and Michalakis, 2007; Biel, 2009). CNGCs are activated by direct binding of cAMP or cGMP to the channel and activation of the channel results in the influx of extracellular cations causing depolarization of the cell membrane. One example of a CNGC is the hyperpolarization-activated and cyclic nucleotide–gated channels (HCN), which are expressed in the heart and the nervous system. These channels mainly conduct Na^+^ and K^+^ and are activated by a hyperpolarized membrane potential and stimulated by intracellular cyclic nucleotides. HCN channels are responsible for the “funny current” (I_f_) in the heart and neurons, where they regulate pacemaker activity and neuronal firing (Difrancesco, 1986; DiFrancesco and Tortora, 1991; Moosmang et al., 1999; DiFrancesco, 2020).

## KCNQ-Encoded Potassium Channels

There are 12 sub-families of voltage-gated potassium channels (Kv1-Kv12), within which the Kv7 potassium channel family consists of five isoforms encoded by the *KCNQ1*-5 genes (Kv7.1-Kv7.5) (Robbins, 2001; Gutman et al., 2005). Like all Kv channels, Kv7 channels are voltage-sensing channels and open and close upon changes in transmembrane potential to selectively let K^+^ ions pass through the channel. The Kv7 channels are comprised of four α-subunits, with each α-subunit consisting of six transmembrane (TM) spanning domains (S1-S6). S1-S4 form the voltage-sensing domain, while S5-S6 form the pore domain. Each α-subunit contains cytoplasmic amino (N) – and carboxyl (C) – termini, which are essential for channel tetramerization and channel regulation (Barros et al., 2012). In the C-terminus of the channel, a coiled-coil sequence motif is the major determinant of channel assembly (Jenke et al., 2003; Schwake et al., 2003, 2006). Assembly of all four α-subunits placed in a cylindrical arrangement creates a pore through which the K^+^ ions are conducted selectively (Heginbotham et al., 1992; Doyle et al., 1998). This assembly can be done with four identical α-subunits from the same Kv7 isoform to form homomeric channels, or by combining at least two types of α-subunits with other Kv7 isoforms to form heteromeric channels. Kv7.1 was thought to only form as a homotetrameric channel (Schwake et al., 2000); however, heterotetrameric assembly with Kv7.5 has been suggested (Oliveras et al., 2014). Kv7.3 homomers are weakly effective but form functional heterotetramers with Kv7.2 (Schroeder et al., 1998; Wang et al., 1998), Kv7.4 (Kubisch et al., 1999) and Kv7.5 (Schroeder et al., 2000a) subunits, and Kv7.4 heterotetramizes with Kv7.5 (Bal et al., 2008; Brueggemann et al., 2014b; Chadha et al., 2014; Jepps et al., 2015). Kv7 channels also interact with KCNE-encoded single transmembrane-spanning auxiliary protein subunits [KCNE1-5; also called MinK and MinK-related peptides (MiRPs)] that function as β or ancillary subunits (Abbott and Goldstein, 1998; McCrossan and Abbott, 2004; Abbott, 2016; Jepps, 2017). These KCNE proteins cannot form functional channels themselves but coassemble with Kv7 channels to modulate several functional properties of the channel, including membrane trafficking, voltage dependence, and kinetics of channel opening and closing (McCrossan and Abbott, 2004; Kanda and Abbott, 2012). Finally, Kv7 channels have a large cytoplasmic C-terminal composed of four segments (helix A-D) (Haitin and Attali, 2008; Xu et al., 2013; Barrese et al., 2018). In addition to coordinating channel tetramerization, several regulatory molecules interact with the C-terminal region to modulate Kv7 channel function, including calmodulin (CaM), phosphatidylinositol 4,5-biphosphate (PIP_2_), protein kinase C (PKC), protein kinase A (PKA), A-kinase-anchoring protein (AKAP), ankyrin G (Ank-G) and Nedd4-2 (summarized by Haitin and Attali (2008) and Barrese et al., 2018).

This review will now focus on the mechanisms of cAMP-dependent regulation of Kv7 channels and explore a possible role for this mechanism for therapeutic targeting in diseases associated with Kv7 channel dysfunction.

## cAMP Regulation of Kv7 Channels

### Kv7.1

Kv7.1 channels are expressed in several tissues throughout the body including the heart, uterus, and epithelial cells of the inner ear, pancreas, airways, gastrointestinal tract and kidneys. In the heart, Kv7.1 associates with the ancillary subunit KCNE1 to constitute the channel responsible for the late repolarizing current termed *I*_Ks_ (Barhanin et al., 1996; Sanguinetti et al., 1996). Kv7.1 also associates with KCNE1 in the inner ear, where they regulate auditory function and pancreatic acinar cells where activation of the Kv7.1-KCNE1 channel provides the driving force for Cl^–^ secretion (Kim and Greger, 1999; Köttgen et al., 1999; Warth et al., 2002). In addition, Kv7.1 channels in the pancreatic ß-cells contribute to the regulation of insulin secretion, and loss of function mutations in the KCNQ1 gene leads to impaired ß-cell insulin secretion, which is associated with type 2 diabetes (Ullrich et al., 2005; Unoki et al., 2008; Yasuda et al., 2008; Yamagata et al., 2011; Liu et al., 2014; Torekov et al., 2014; Min Lee et al., 2017; Zhang et al., 2020). Furthermore, association of Kv7.1 with KCNE3 in epithelial tissues of the colon, small intestine and airways regulates the transport of water and salts (Schroeder et al., 2000b; Grahammer et al., 2001a, b; Vallon et al., 2005).

Kv7.1-KCNE1 channels have been studied extensively in cardiac myocytes where the channel is important for delayed cardiac repolarization and shapes action potential duration (Terrenoire et al., 2005). In addition, it is enhanced by sympathetic nerve activity through activation of β-adrenergic receptors (Bennett and Begenisch, 1987; Yazawa and Kameyama, 1990). Stimulation of the β-adrenergic receptor by noradrenaline released from the cardio-accelerator nerve causes an elevation of cAMP that increases *I*_Ks_ via PKA-dependent phosphorylation (Yazawa and Kameyama, 1990).

**FIGURE 1:**
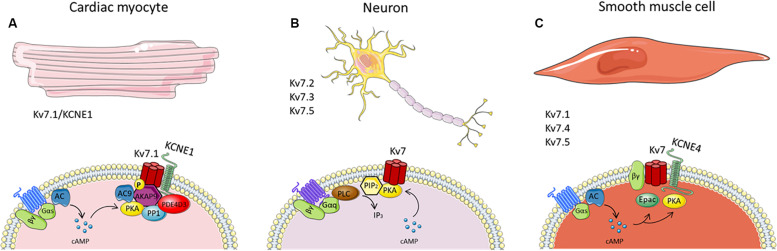
Schematics of known cAMP-dependent Kv7 channel regulation in panel cardiac **(A)**, neurones **(B)** and smooth muscle cells **(C)**. The figure summarizes many of the known interaction partners involved in the cAMP-mediated regulation of Kv7 channels in the different cells types, thereby highlighting the heterogeneity of cAMP-Kv7 channel signaling. It should be noted that, although AKAP79/150 is associated with Kv7.2 channels, it has not been included in the figure since there is no evidence of its involvement in PKA phosphorylation of the channel.

As mentioned previously, PKA actions are regulated by AKAPs, by positioning PKA and other cAMP-responsive enzymes in proximity to their substrates. In the heart, several AKAPs are expressed, including AKAP79/150 (human AKAP79/murine AKAP150), AKAP15/18δ and AKAP9. AKAP9, better known as yotiao, facilitates PKA-phosphorylation of serine 27 in the Kv7.1 N-terminus required for the proper functioning of the channel (Walsh et al., 1988; Walsh and Kass, 1991; Potet et al., 2001; Marx et al., 2002). Yotiao also associates with protein phosphatase 1 (PP1), phosphodiesterase (PDE) and AC to control *I*_Ks_ phosphorylation status (Terrenoire et al., 2009; Li et al., 2012). Yotiao binds directly to Kv7.1 C-terminus via a leucine zipper motif, an amino acid sequence in the C terminus of Kv7.1, and disruption of the complex by mutations in yotiao or KCNQ1 are associated with Long QT Syndrome (Kass et al., 2003; Fodstad et al., 2004; Chen et al., 2007). Besides acting as a scaffold protein, yotiao has a functional role that occurs after *I*_Ks_ phosphorylation. Studies revealed that cAMP-dependent activation of Kv7.1-KCNE1 depends not only on serine 27 phosphorylation but also requires the direct binding of yotiao to the Kv7.1 at the leucine zipper motif (Kurokawa et al., 2004; Chen et al., 2005; Chen and Kass, 2006). Furthermore, it was revealed that yotiao itself is a substrate for PKA phosphorylation and can be phosphorylated upon β adrenoceptor stimulation (Chen et al., 2005). Serine 43 was identified as target residue for PKA phosphorylation on yotiao, with mutations of this residue dampening cAMP effects on the *I*_Ks_ current, even though yotiao binding and phosphorylation of Kv7.1 channels were not affected. Taken together, these studies conclude that AKAP protein yotiao has a crucial and complex regulatory role on the cAMP-dependent activity of *I*_Ks_ channels (see Figure 1A).

Although PKA activation of Kv7.1 following β adrenergic stimulation can increase *I*_Ks_, chronic β adrenergic stimulation downregulates the *I*_Ks_ response (Mona et al., 2014). Chronic β adrenergic stimulation led to a significant decrease in KCNE1 but not KCNQ1 mRNA expression in guinea pig cardiomyocytes. Furthermore, chronic *in vitro* and *in vivo* isoprenaline stimulation reduced KCNE1 protein expression and KCNE1 membrane expression in guinea pig cardiomyocytes. This effect was mediated via Epac1 and not PKA activation, leading to increased translocation of nuclear factor of activated T cell (NFAT), which was responsible for KCNE1 downregulation (Mona et al., 2014).

In the inner ear, cAMP activates Kv7.1-KCNE1 channels, which results in the secretion of K^+^, necessary for normal hearing (Wang et al., 1996; Neyroud et al., 1997). However, the precise mechanisms underlying the cAMP-regulated increase in K^+^ conductance in the inner ear are unclear (Sunose et al., 1997). In the intestine, cAMP enhances Kv7.1-KCNE3 currents and stimulates Cl^–^ secretion by hyperpolarizing the cell membrane and thereby amplifying the driving force for Cl^–^ exit through cystic fibrosis transmembrane conductance regulator Cl^–^ channels (Lohrmann et al., 1995; Diener et al., 1996; Suessbrich et al., 1996; Devor et al., 1997; Rufo et al., 1997; Schroeder et al., 2000b; Bajwa et al., 2007). An essential role for KCNE3 in cAMP-driven Cl^–^ secretion has been suggested from the observation that KCNE3 knockdown reduced cAMP-mediated Cl^–^ secretion across tracheal and intestinal epithelia without altering Kv7.1 expression (Preston et al., 2010) but the precise mechanism by which cAMP stimulates Kv7.1-KCNE3 channels in these cells is still unknown. Like for intestine epithelial cells, airway epithelial cells secrete Cl^–^ stimulated by the cAMP-signaling pathway, where blockade of Kv7.1 channels suppress the cAMP-mediated Cl^–^ secretion (Mall et al., 2000; Grahammer et al., 2001b; MacVinish et al., 2001; Cowley and Linsdell, 2002; Kim et al., 2007). However, the direct mechanism how cAMP mediates Kv7.1 channel activation responsible for Cl^–^ secretion needs to be further investigated. In pancreatic β-cells, Kv7.1 channels contribute to insulin secretion. Besides, a link between cAMP and insulin secretion has established (Malaisse and Malaisse-Lagae, 1984; Seino and Shibasaki, 2005). However, it remains to be determined if Kv7.1 channels play a role in this cAMP-dependent mechanism.

### Modifiers of cAMP-Mediated Regulation of Kv7.1 Channels

Although the direct regulation of cAMP signaling on Kv7.1 channel activity is well described, several factors can modulate this interaction indirectly. For instance, Nicolas et al. (2008) determined that the PKA-dependent regulation of *I*_Ks_ was microtubule-dependent (Nicolas et al., 2008). Cytoskeletal microtubules are essential for proper trafficking of cardiac ion channels to the plasma membrane (Steele and Fedida, 2014), and although a physical interaction between Kv7.1 and the microtubule forming protein, β-tubulin, was shown, disruption of the microtubules did not modify the Kv7.1-KCNE1 channel membrane density or baseline currents in transfected COS-7 cells or cardiomyocytes. However, microtubule disruption decreased the *I*_Ks_ response to PKA-mediated stimulation. This was not due to altered channel phosphorylation, yotiao phosphorylation or the interaction between both proteins, suggesting that microtubules play an important role in the coupling of PKA-dependent Kv7.1 phosphorylation and its channel activation (Nicolas et al., 2008).

The effect of cAMP on the trafficking of Kv7.1 channels has also been investigated. PKA inhibition in Madin-Darby canine kidney (MDCK) cells reduces Kv7.1 channel membrane expression and increases intracellular accumulation of the Kv7.1 protein in late endosomes/lysosomes (Andersen et al., 2015). This suggests a role for PKA-mediated trafficking of Kv7.1 channels, although this was not a result of channel phosphorylation. A previous study by the same group found that two cAMP phosphorylation residues on Kv7.1, serine 27 and serine 92, were not crucial for trafficking Kv7.1 (Lundby et al., 2013). Instead the E3 ubiquitin ligase Nedd4-2 is required in this PKA-dependent trafficking pathway (Andersen et al., 2015). Nedd4-2 is an ubiquitin-protein ligase that binds to ion channels containing a C-terminal proline-rich segment (PY motif) (Manning and Kumar, 2018). Among the Kv7 channels, Kv7.1 is the only isoform containing this sequence motif. Binding of Nedd4-2 to the C-terminal PY motif in Kv7.1 channels regulates the ubiquitylation of the channel and its internalization (Jespersen et al., 2007). Andersen et al. (2015) concluded that PKA regulated Nedd4-2-dependent trafficking of Kv7.1 but the precise mechanism how PKA influences Nedd4-2 needs to be determined (Andersen et al., 2015).

### Kv7.2 and Kv7.3

Kv7.2 and Kv7.3 channels are widely expressed in neuronal tissues, where they form a heteromeric channel that generates the “M-current” (Brown and Adams, 1980; Wang et al., 1998; Wickenden et al., 2001; Roche et al., 2002; Shah et al., 2002). Kv7.5 channels are also a component of the M-channel in heteromeric formation with Kv7.3 (Lerche et al., 2000; Schroeder et al., 2000a; Tzingounis et al., 2010). The M-current in neurons maintains the negative resting membrane potential to limit neuronal firing and excitability (Marrion, 1997; Jentsch, 2000). M-currents are inhibited by Gq/11 coupled GPCRs such as muscarinic acetylcholine receptors M1, M3 and M5, or bradykinin B2 receptors. Stimulation of these receptors decreases M-current density resulting in depolarization, which subsequently increases neuronal excitability (Brown and Selyanko, 1985; Cruzblanca et al., 1998). It is well established that disruption of the M-current due to mutations in either *KCNQ2* or *KCNQ3* genes causes excessive neuronal excitability, leading to neuronal diseases, such as benign familial neonatal convulsions and epileptic encephalopathy (Biervert et al., 1998; Charlier et al., 1998; Singh et al., 1998; Jentsch, 2000; Castaldo et al., 2002; Borgatti et al., 2004; Weckhuysen et al., 2012, 2013).

Kv7.2/Kv7.3 currents in overexpressed *Xenopus* oocytes are enhanced by cAMP, which relies on PKA-mediated phosphorylation of serine 52 in the Kv7.2 N-terminus (Schroeder et al., 1998). Furthermore, Cooper et al. (2000) identified an interaction of Kv7.2 and PKA subunits in human brain samples by co-immunoprecipitation and affinity chromatography (Cooper et al., 2000). In addition, AKAP79/150 is associated with Kv7.2 channels (Hoshi et al., 2003, 2005; Zhang and Shapiro, 2012). Although the core AKAP79/150 complex contains PKA (Gold et al., 2006), it has not yet been determined whether AKAP70/150 facilitates PKA phosphorylation of Kv7.2 channel; however, AKAP79/150 is essential for the recruitment and phosphorylation of Kv7.2 channels by PKC (Hoshi et al., 2003; Higashida et al., 2005; Zhang and Shapiro, 2012).

Multiple other phosphorylation sites of Kv7.2/Kv7.3 channels have been identified using mass spectrometry. However, the responsible kinase for this phosphorylation remains elusive as not only PKA but also PKC and src tyrosine kinase can regulate Kv7.2/Kv7.3 channel phosphorylation (Gamper et al., 2003; Hoshi et al., 2003; Li et al., 2004; Surti et al., 2005). A recent study by Salzer et al. (2017) found 13 phosphorylation sites for human Kv7.2 using mass spectrometry, one already identified (serine 52) located at the N-terminus, whereas the remaining 12 were located in the C-terminus. Using *in vitro* phosphorylation assays the authors identified the protein kinases responsible for C-terminus Kv7.2 phosphorylation. Only two of the 12 residues (serine 438 and serine 455) were phosphorylated by PKA. Inhibition of PKA reduced Kv7.2 phosphorylation, which decreased channel sensitivity to PIP_2_ depletion, thereby attenuating Kv7 channel regulation via M1 muscarinic receptors. Thus, phosphorylation of the Kv7.2 channel is necessary to maintain a reduced affinity for PIP_2_ (Salzer et al., 2017; Figure 1B).

Kv7.5 channels are expressed in some regions of the brain, where they coassemble with Kv7.3 channels. Kv7.5/Kv7.3 channels show similar currents to the Kv7.2/Kv7.3 currents and are also inhibited by M1 muscarinic receptor activation (Schroeder et al., 2000a). Kv7.5 can be phosphorylated and stimulated by PKA on serine 53 on the N-terminus (Brueggemann et al., 2018); however, it is still unclear whether the heterotetrameric Kv7.3/Kv7.5 channel found in neurones are regulated in the same way by cAMP.

### Kv7.4 and Kv7.5

Kv7.4 channels are expressed in the inner ear and auditory nerves (Kharkovets et al., 2000), the substantia nigra (Hansen et al., 2008), skeletal muscle cells (Iannotti et al., 2010), the mitochondria of cardiac myocytes (Testai et al., 2016) and in smooth muscle cells extensively (see Barrese et al., 2018 for a recent review of the smooth muscle literature). Kv7.5 channels are expressed in the brain, where they play a role in the regulation of neuronal excitability, and in skeletal and smooth muscle (Schroeder et al., 2000a; Roura-Ferrer et al., 2008; Iannotti et al., 2010; Haick and Byron, 2016).

In smooth muscle, there is evidence Kv7.4 and Kv7.5 channels exist as both homomers and heteromers (Brueggemann et al., 2011, 2014b; Chadha et al., 2014; Jepps et al., 2015). The first indication that smooth muscle Kv7 channels were activated by cAMP came from gastric smooth muscle cells. In these cells, β adrenoceptor stimulation with isoprenaline induced a current that resembled the neuronal M current, although at this time the molecular identity of the M current was still unknown (Sims et al., 1988).

In vascular smooth muscle, the use of pharmacological inhibitors and molecular interference has identified a role for Kv7 channels in receptor-mediated relaxations. The first identification was for β adrenoceptor-mediated vasodilatation in the renal vasculature (Chadha et al., 2012). Inhibition of Kv7 channels by XE991 or linopirdine and knockdown of Kv7.4 with *KCNQ4*-targeted small interfering RNA (siRNA), attenuated arterial relaxations to the β adrenoceptor agonist, isoprenaline (Chadha et al., 2012). More recent, experiments with morpholino-mediated suppression of Kv7.4 translation corroborated these findings (Stott et al., 2018). Furthermore, isoprenaline increased Kv7 currents in vascular smooth muscle cells isolated from rat arteries and in A7r5 cells (a rat aortic smooth muscle cell line) (Chadha et al., 2012; Stott et al., 2015; Mani et al., 2016; Brueggemann et al., 2018).

In hypertension, Kv7.4 protein expression is diminished causing reduced hyperpolarization and relaxation of the smooth muscle cells. Furthermore, in renal arteries from spontaneously hypertensive rats, where Kv7.4 expression is reduced, receptor-mediated relaxations were diminished (Jepps et al., 2011; Chadha et al., 2012). Interestingly, in the cerebral arteries from hypertensive rats, there is no compromise of Kv7.4 channel expression and CGRP receptor-mediated relaxations are unaffected (Chadha et al., 2014).

Although targeting Kv7.4 in isolated vessels with siRNA- and morpholino-induced knockdown inhibits isoprenaline- and CGRP-induced relaxations (Chadha et al., 2014; Stott et al., 2018), there is evidence that Kv7.5 is required to elicit cAMP-dependent relaxations. In A7r5 cells, stimulation of β adrenoceptors and the downstream pathway enhanced the currents produced by Kv7.5 channels and heterotetrameric Kv7.4/Kv7.5 channels expressed exogenously, but not when homomeric Kv7.4 channels were expressed (Mani et al., 2016), suggesting cAMP-mediated regulation of Kv7.4 and Kv7.5 depends on the stoichiometry of the channel.

The precise Kv7 channel stoichiometry eliciting cAMP-dependent relaxations in vascular smooth muscle is still unknown. Kv7.4 channels are important for proper Kv7 channel function in arteries, highlighted by their downregulation in arteries from hypertensive animals, whereas both Kv7.4 and Kv7.5 may play a role in the cAMP-dependent activation. To further complicate this mechanism, KCNE4 co-assembles with Kv7.4 and Kv7.5 in vascular smooth muscle cells to alter the biophysical properties and cellular localization of these channels. Targeted knockdown of *Kcne4* in rat arteries depolarized the smooth muscle resting membrane potential and reduced vasorelaxations to Kv7 channel activators. Interestingly, in *Kcne4* knockout mice, only the males displayed attenuated Kv7 channel function, but both male and female *Kcne4* knockout mice had attenuated responses to isoprenaline. Given that *Kcne4* expression has been shown in several arterial beds, it could be facilitating the Kv7 channel-dependent cAMP relaxations (Jepps et al., 2015; Abbott and Jepps, 2016). The expression of KCNE4 in A7r5 cells is yet to be determined but including KCNE4 subunits in future studies investigating the cAMP-dependent Kv7.4/Kv7.5 channel activation could give valuable insight into this mechanism.

Both PKA and Epac enhance Kv7 channel activity in vascular smooth muscle (Khanamiri et al., 2013; Mani et al., 2016; Stott et al., 2016). Isoprenaline-mediated relaxations were attenuated by PKA inhibition in renal arteries, whilst the isoprenaline relaxations in mesenteric arteries were not affected (Stott et al., 2016). In the mesenteric artery, isoprenaline-induced linopirdine-sensitive relaxations were elicited through activation of Epac, which is present in both rat renal and mesenteric arteries, but more predominant in mesenteric arteries. With electrophysiology experiments, Kv7 currents were increased with direct Epac stimulation in smooth muscle cells isolated from renal and mesenteric arteries (Stott et al., 2016). These data highlight that cAMP-dependent stimulation of Kv7 channels in vascular smooth muscle is not only dependent on the Kv7 channel architecture but also the channel’s association with downstream cAMP effector proteins, all of which are likely to be artery specific (see Figure 1C).

Kv7 channels are also expressed in airway smooth muscle cells (ASMCs) of rodents and humans. Pharmacological activators of Kv7 channels, including retigabine, flupirtine and S-1, relax airway smooth muscle cells from guinea pigs and human, and are important regulators of airway diameter (Brueggemann et al., 2012; Evseev et al., 2013). β2-adrenergic receptor agonists are commonly used for the treatment of asthma to relieve hyperconstriction of the airway (Cazzola et al., 2013), and are able to enhance Kv7 currents in ASMCs thereby inducing relaxation of rat airways (Brueggemann et al., 2014a). A later study by Brueggemann et al. (2018) revealed that β adrenoceptor stimulation activated the Kv7.5 channels in cultured human ASMCs, a response which was mediated by the PKA pathway (Brueggemann et al., 2018). This study identified serine 53 on the Kv7.5 N-terminus to be responsible for the increased Kv7.5 currents in response to cAMP elevation, which could be a common mechanism for β adrenoceptor/cAMP activation of Kv7.5 channels in vascular smooth muscle cells. Recent findings show that phosphorylation of serine 53 on the N-terminus of Kv7.5 increases its affinity for PIP_2_, corresponding to enhanced channel activation (Brueggemann et al., 2020).

Kv7.4 and Kv7.5 are also expressed in the smooth muscle of the gastrointestinal tract (Ohya et al., 2002; Jepps et al., 2009; Ipavec et al., 2011; Adduci et al., 2013), bladder (Streng et al., 2004; Rode et al., 2010; Svalø et al., 2012, 2013; Afeli et al., 2013; Anderson et al., 2013), penis (Jepps et al., 2016) and uterus (McCallum et al., 2009) where important functional roles have been established. Whether Kv7 channels in these tissues are subjected to the same cAMP-dependent regulation as in vascular and airway smooth muscle remains to be determined. Given that, even within the vasculature, there are differences in the cAMP-dependent mechanisms that lead to Kv7 channel stimulation, the Kv7 channels in these smooth muscle tissues cannot be assumed to behave in the same way as has been described in airway and vascular smooth muscle. Future work should investigate how Kv7 channels are subjected to cAMP-dependent regulation in these tissues, which could lead to novel therapeutic strategies for the treatment of various smooth muscle disorders, such as constipation, preeclampsia, erectile dysfunction and incontinence.

### Modifiers of cAMP-Mediated Regulation of Kv7.4 Channels

Gβγ subunits have a regulatory role on the modulation of Kv7.4 channels in vascular smooth muscle cells, which is Gβ subunit-specific (Stott et al., 2015, 2018; Greenwood and Stott, 2020). Gβγ subunit binding to Kv7.4 channels enhances the current in both HEK and CHO cells expressing Kv7.4, and in rat renal arterial smooth muscle cells. In addition, Gβγ subunit interaction is critical for the β adrenergic dependent activation of Kv7 channels in rat renal arteries, but not in rat mesenteric arteries. Contrarily, inhibition of Gβγ subunits attenuated the CGRP dependent relaxations in mesenteric arteries, but showed no effect on CGRP relaxation in cerebral arteries, highlighting the role of Gβγ in Kv7-dependent vasorelaxations to be artery specific and dependent on the vasorelaxant (Stott et al., 2018).

The microtubule network has also been implicated in the Kv7 channel-dependent cAMP-mediated relaxations in rat mesenteric and renal arteries (Lindman et al., 2018). Disruption of the microtubule network with colchicine increased the membrane expression of Kv7.4 channels, which enhanced membrane hyperpolarization, decreases in intracellular Ca^2+^ and vasorelaxation to isoprenaline and forskolin in a Kv7 channel-dependent manner, specifically (Lindman et al., 2018). Further work in this area is required to determine whether the microtubule network regulates cAMP signaling in smooth muscle cells.

## Conclusion

The second messenger cAMP regulates Kv7 channel activity in many cell types, including cardiac myocytes, smooth muscle and neurons. However, cAMP-mediated regulation of Kv7 channels is complex and many aspects remain to be elucidated. Generation of cAMP results from stimulation of AC, of which 9 isoforms exist. Depending on the isoforms expressed in a cell type, extracellular signals through GPCR activation can be integrated differently. Identifying how different AC isoforms contribute to cAMP-dependent activation of Kv7 channels in different tissue types could lead to targeting of a particular isoform providing therapeutic benefits with fewer adverse effects. Furthermore, the recent discovery of Epac activation of Kv7.4/Kv7.5 channels in vascular smooth muscle adds more complexity to this pathway. Determining whether this cAMP/Epac pathway plays a role in the activation of other Kv7 channels will be necessary to fully understand the physiological impact of cAMP signaling through Kv7 channels. Finally, the importance of the KCNE subunits in the cAMP-dependent regulation of Kv7 channels has often been overlooked but is likely to have a substantial role. By better defining the cAMP regulation of Kv7 channels in different tissues, it may become possible to exploit subtle tissue-specific differences in the activation pathways in order to generate novel therapeutics.

## Author Contributions

All authors contributed to the drafting, revision and final approval of the manuscript.

## Conflict of Interest

The authors declare that the research was conducted in the absence of any commercial or financial relationships that could be construed as a potential conflict of interest.
